# Investigating the Interplay Between Matrix Compliance and Passaging History on Chondrogenic Differentiation of Mesenchymal Stem Cells Encapsulated Within Alginate-Gelatin Hybrid Hydrogels

**DOI:** 10.1007/s10439-023-03313-y

**Published:** 2023-07-15

**Authors:** Mohamad Orabi, Gargi Ghosh

**Affiliations:** 1 Department of Mechanical Engineering, University of Michigan, Dearborn, 4901 Evergreen Road, Dearborn, MI 48128 USA; 2 Amgen Bioprocessing Center, Henry E. Riggs School of Applied Life Sciences, Keck Graduate Institute, 535 Watson Drive, Claremont, CA 91711 USA

**Keywords:** Mesenchymal stem cells, Alginate, Gelatin, Chondrogenic differentiation, Matrix mechanics

## Abstract

Mesenchymal stem cells (MSCs) are used widely in tissue engineering and regenerative medicine because of their ease of isolation and their pluripotency. The low survival and retention rate of MSCs at the target site upon implantation can be addressed via encapsulation within hydrogels capable of directing their fate. In this study, the interplay between matrix mechanics and the passage number of MSCs on their chondrogenic differentiation was assessed. Human bone marrow-derived MSCs between passages 4 and 6 were encapsulated within alginate-gelatin hybrid gels. The stiffness of the gels was varied by varying alginate concentrations while maintaining the concentration of gelatin and consequently, the cell adhesion sites, constant. The study revealed that within 4.8 kPa gels, GAG deposition was higher by P4 MSCs compared to P6 MSCs. However, an opposite trend was observed with collagen type 2 deposition. Further, we observed enhanced chondrogenic differentiation upon encapsulation of MSCs within 6.7 kPa hydrogel irrespective of passaging history. However, the effect of matrix compliance was more prominent in the case of higher passaged MSCs suggesting that matrix stiffness can help rescue the reduced differentiation capability of these cells.

**Supplementary Information:**

The online version contains supplementary material available at 10.1007/s10439-023-03313-y.

## Introduction

Mesenchymal stem cells (MSCs) have been used widely in tissue engineering and regenerative medicine [1]. They are well known for their easy isolation, high proliferation capacity, and multipotency [2, 3]. Their ability to differentiate into specialized cells made them one of the dominant resources for repairing tissue injuries and defects [4]. However, the clinical translation of MSC-based therapy is limited by low engraftment and retention rate of cells at the target sites [5], thereby highlighting the need for strategies to address this challenge. Biomaterial-based approaches permit enhanced control of cells as well as play a critical role in stem cell fate via regulation of cell adhesion, proliferation, matrix production, and organization into functional tissues [6]. Cell encapsulation technology is the most prominent way to immobilize stem cells in a permeable matrix such as hydrogels [7]. Hydrogels are widely used in tissue engineering as they swell in presence of an aqueous solution but are insoluble in water [8]. The cross-linked 3D microstructures conserve the elastic properties of soft tissues and provide the proper micro-environment for cells to grow [7].

Cartilage is a tough and flexible connective tissue that provides a cushion between bones in the joints. Unlike other connective tissues, cartilage is not vascularized. It is composed primarily of chondrocytes, collagen, proteoglycans, and water that produce and maintain a structural matrix giving cartilage tissue its form and function [9]. Different cell resources such as chondrocytes and stem cells are used for the regeneration of cartilage tissue [10, 11]. Chondrocytes isolated from patient cartilage biopsy are expanded *ex vivo* and used for transplantation. The isolated chondrocytes have the advantage of a high level of matrix secretion and lack hypertrophy [12]. However, clinical trials highlighted that the major drawback of using autologous chondrocytes involves chondrocyte dedifferentiation during in vitro expansion [13]. Besides chondrocytes, stem cells such as bone marrow-derived mesenchymal stem cells (MSCs) and adipose-derived MSCs have been identified as useful cell resources for the regeneration of cartilage tissue [7]. The regeneration was found to be more effective upon utilization of porous scaffolds that encourage the diffusivity of oxygen and nutrients [14, 15].

The effect of different collagen types as well as different scaffold properties including pore diameters on the behavior of chondrocytes was studied and collagen type 2 was reported to stabilize the chondrocyte morphology and glycosaminoglycans synthesis [16]. Studies reported that type 2 collagen promoted the chondrogenic differentiation of MSCs [17]. However, the major drawback of type 2 collagen is the limited safety approvals due to its arthritogenic activity [18, 19]. Alginate is widely used in tissue engineering for its biocompatibility, mechanical properties, and fast gelation kinetics [20–23]. It is commonly used for cellular encapsulation to protect the cells from the host’s immune system as well as to improve the retention of cells at the target site. However, in the absence of cell-binding sites, alginate scaffolds lead to poor adhesion of cells. Gelatin, due to its biocompatibility, lack of immune responses, biodegradability, and presence of the cell adhesive sites of collagen, is one of the most often used materials for cell culture [24]. The combination of alginate and gelatin, therefore, is excellent for 3D cell culture. Earlier studies have indicated that encapsulating MSCs in alginate gelatin hydrogels provided the proper microenvironment for stem cells to proliferate and differentiate [25]. It is, however, not well understood whether matrix properties preferentially permit MSCs of certain passage numbers to acquire a phenotype favorable for cartilaginous matrix deposition. To the best of our knowledge, the interplay between alginate-gelatin composition/stiffness and the passaging history of MSCs on chondrogenic differentiation has not been studied. For this purpose, bone marrow derived human MSCs (through passages 4 to 6) were encapsulated in 1.5%, 2.0%, 2.5%, and 5.0% (w/v) alginate while gelatin concentration was maintained at 5.0% (w/v). The impact of hydrogel composition/stiffness on the metabolic activity and chondrogenic differentiation, as manifested from cartilaginous matrix deposition, was explored as a function of MSC passaging history.

## Materials and Methods

### Materials

Alginic acid sodium salt (Algin* Sodium alginate powder), gelatin from porcine skin (powder, gel strength − 300 g Bloom, Type A, BioReagent, for electrophoresis, suitable for cell culture), 50 µg/ml fluorescein isothiocyanate (FITC)-dextran (150,000 kDa), and calcium chloride (anhydrous, granular, < 7.0 mm, > 93.0%) were procured from SIGMA ALDRICH (St. Louis, MO, USA). Dulbecco’s Phosphate Buffered Saline (DPBS), Dulbecco’s Modified Eagle Medium (DMEM), Penicillin Streptomycin (Pen Strep), 0.25% Trypsin-EDTA, Fetal Bovine Serum (FBS), and collagenase type I were acquired from Gibco by Life Technologies (Grand Island, NY). Hoechst 33342, methanol, acetone, and cell viability assay kit were obtained from Thermofisher Scientific (Waltham, MA). XTT Cell Proliferation Assay Kit was purchased from ATCC (Manassas, VA). Chondrogenic cell basal medium and Glycosaminoglycans Assay Kit were obtained from Lonza (Walkerville, MD) and Chondrex Inc. (Washington), respectively. Anti-human collagen type 2, mouse, purified IgG antibody was purchased from MP Biomedicals. Anti-human aggrecan monoclonal antibody, mouse, purified IgG1 antibody, and Alexa Fluor 488 goat anti-mouse IgG (H + L) were procured from Thermofisher Scientific. Safranin O (crystalline powder) was purchased from Alfa Aesar.

### Preparation of Alginate-Gelatin Solutions

Alginate-gelatin hydrogel was prepared as described previously [26]. Alginic acid sodium salt powder (Sigma Aldrich) was added to distilled water and stirred continuously at room temperature until fully dissolved to prepare 1.5%, 2.0%, 2.5%, and 5.0% (w/v) alginate solutions. To prepare the alginate-gelatin solution, gelatin type A powder (from porcine skin, Sigma Aldrich) was added to Dulbecco’s Phosphate Buffer Saline solution (DPBS, Gibco by Life Technologies) and stirred continuously at 60 °C until fully dissolved to prepare a 5.0% (w/v) gelatin solution. Alginate powder was then added to the 5.0% (w/v) gelatin solution to generate solutions with varying alginate concentrations while maintaining gelatin concentration constant.

### Preparation of Alginate-Gelatin Microspheres

**Fig. 1:**
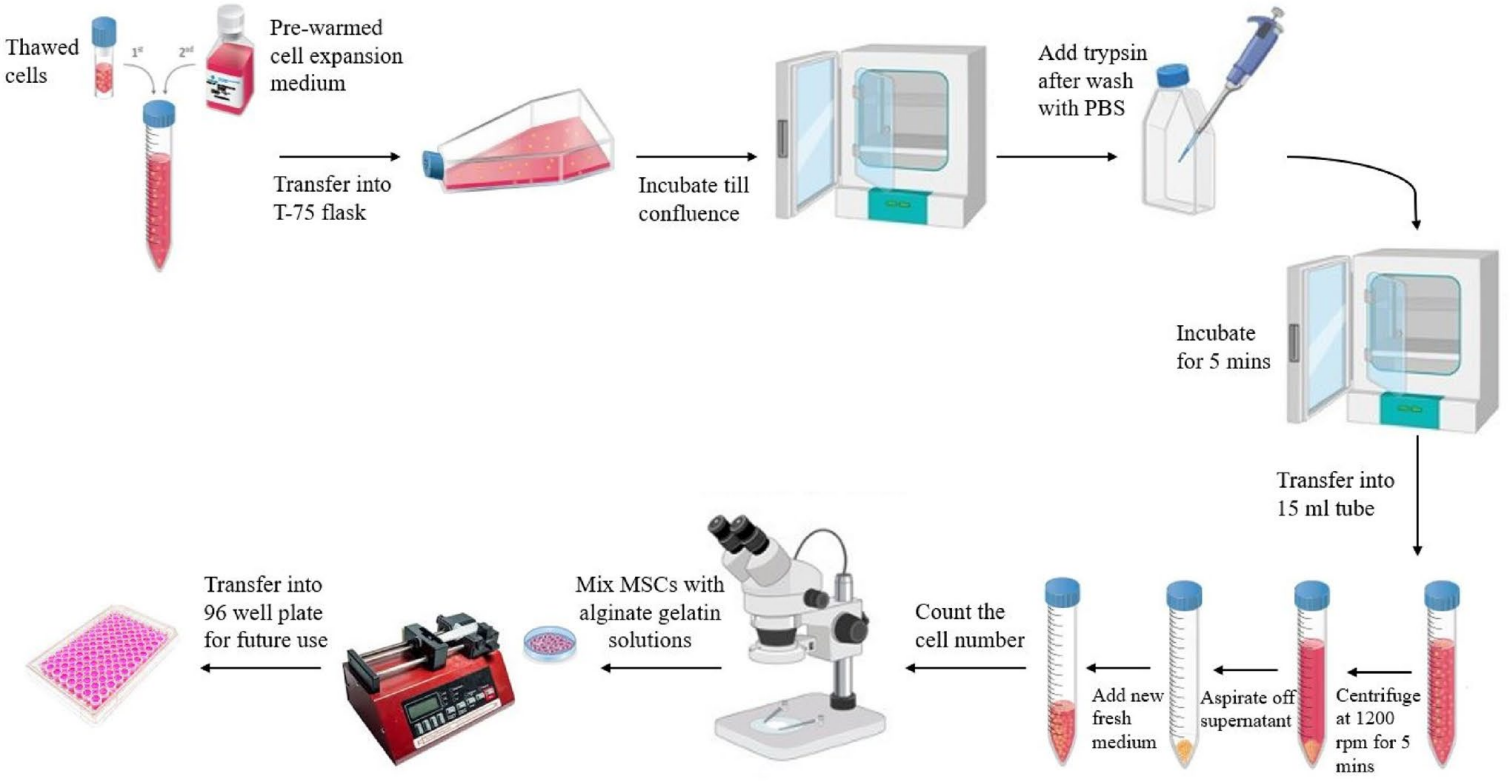
Illustration of preparation of encapsulated alginate-gelatin gels with MSCs.

The alginate ± gelatin microspheres were generated using a 3 ml syringe with a BD Precision Glide needle of 30G coupled to a syringe pump (Legato 270, KD Scientific, USA). The solution (alginate or alginate-gelatin) was pumped at a flow rate of 1 ml/min into 5.0 ml of 0.5 M CaCl_2_ (Sigma Aldrich) solution. The microspheres were incubated under stirred condition (60 rpm) at 37 °C for 10 min, following which they were washed using Dulbecco's Phosphate Buffered Saline (DPBS, Gibco by Life Technologies) and transferred to 96 well plates for further experiments (Fig. 1).

### Characterization of Hydrogels

#### Swelling Ratio

To measure swelling ratio (SR), the hydrogel microspheres were fabricated as described earlier and incubated for 48 h in DPBS (Gibco by Life Technologies) on a rotator shaker at 50 rpm at room temperature. Following incubation, the excess solutions from the surfaces were removed with Kim wipes and then the weights of the hydrated samples were measured (W_wet_). The hydrogels were then dried at 50 °C, and their dry weights (W_dry_) were measured after 24 h. The swelling ratio of the hydrogels was then calculated using the following equation:

1 $$SR= \frac{{W}_{wet}}{{W}_{dry}}$$

#### Degradation

To measure the rate of hydrogel degradation, the alginate or hybrid microspheres were initially incubated in DPBS for 30 mins for equilibration prior to recording the initial weights of the gels (day 0). Then, the samples were incubated in 2.5 units/ml collagenase type I (Gibco by Life Technologies) solution on a rotator shaker at room temperature. The weights of the hydrated hydrogels were recorded each day for 8 consecutive days. The degradation ratio was determined by comparing the weights of the hydrated samples on each day to the weights of the samples on the day of hydrogel fabrication.

#### Diffusion

To assess the transport of macromolecules across the gels, 50 µg/ml fluorescein isothiocyanate (FITC)-dextran (150,000 kDa, Sigma Aldrich, USA) was added to alginate as well as alginate-gelatin solutions prior to gelation. The pre-polymer solutions were placed in cylindrical molds and gelled as described earlier. 500 µl of DPBS was then added to each hydrogel and samples were placed on a shaker. DPBS was then collected after 1, 3, 5, 7, and 8 days and replaced with fresh DPBS. Collected DPBS were analyzed using a SpectraMax M3 fluorescence spectrometer.

To determine the mechanism of dextran diffusion through the hydrogels, the release of macromolecules was fitted into the Korsmeyer-Peppas model given by the following equation:

2 $$\mathrm{F}= \frac{{\mathrm{M}}_{t}}{{\mathrm{M}}_{0}}= {\mathrm{k}}_{1}{\mathrm{t}}^{n}$$

where F is the fractional release of the molecule, M_t_ is the amount of dextran released at any time point, M_0_ is the total mass of dextran that was encapsulated within the initial hydrogels, k_1_ is the kinetic constant, t is the release time, and n is the diffusional exponent. For n ≤ 0.5, the transport of macromolecules is regulated by diffusion, 0.5 ≤ n ≤ 0.9 both by diffusion as well as polymer relaxation/erosion, and for n ≥ 0.9, the transport of molecules is governed via polymer chain relaxation/erosion. The data fitting was carried out on the first 60% cumulative release when the ratio is 0.6. The effective diffusivity (D, cm^2^/s) of the molecules is related to the cumulative release by the following equation:

3 $$\frac{{{\text{M}}_{t} }}{{{\text{M}}_{0} }} = 4*\left( {\frac{{{\text{Dt}}}}{{2{\pi L}}}} \right)^{2}$$

where L is the thickness (cm). The diffusivity can then be related to Korsmeyer-Peppas constants:

4 $${\text{D}} = {\pi L}^{2} \left( {\frac{{{\text{k}}_{1} }}{4}} \right)^{\frac{1}{n}}$$

#### Compressive Modulus/Stiffness

To measure the stiffness of the hydrogels, the pre-polymer solutions were placed in cylindrical molds measuring 10 mm in diameter and approximately 3 mm in thickness and gelled using CaCl_2_ as described earlier in this study. The hydrogels were incubated in DPBS (Gibco by Life Technologies) for 48 h before being compressed directly using a uniaxial testing machine (Test Resources, USA) at a loading rate of 1.2 mm/min and a precision load of up to 4.9 N. At least 3 samples per group were used for each test. Maximum strain and stress at the moment of fracture were recorded, and the compression modulus was calculated from the initial 10% compression [27].

### Cell Culture

Mesenchymal stem cells (MSCs) derived from bone marrow of a healthy male donor, 37 years of age were purchased from LONZA (Morrisville, NC) and expanded in Dulbecco’s Modified Eagle Medium (DMEM) supplemented with 10% (v/v) fetal bovine serum (FBS) and 1% (v/v) penicillin streptomycin. The cells were cultured and incubated until confluence at 37 °C and 5% CO_2_. In this study, cells up to passage 6 were used.

#### Metabolic Activity of the Cells

MSCs were added to either alginate or alginate-gelatin hybrid pre-polymeric solutions at a density of 4.3 × 10^5^ cells/ml and cell-laden hydrogels were fabricated as described earlier. After fabrication, the microspheres were then washed with DPBS, and 10 microspheres were transferred to each well of 96 well plates and incubated with 200 µl of DMEM or chondrogenic media (composed of chondrogenic basal medium and hMSC chondrogenic singlequots^TM^ kit supplement (Walkerville, MD)) at 37 °C and 5% CO_2_. On days 3, 7, 10, 15, and 21 days, activated XTT reagent (100 µl of 10 µM tetrazolium dye, XTT reagent, and 2 µl of 1 µM activation reagent (ATCC, Manassas, VA)) was added to the cells along with fresh media and incubated at 37 °C and 5% CO_2_ for 4 h. The absorbance was then measured using a BioTek Eon Microplate reader at a wavelength of 450 nm. The metabolic activities were calculated as normalized absorbance with respect to day 3. The data were collected from at least three independent experiments, each of which was carried out in three replicates.

#### Cell Viability

Viability of MSCs encapsulated in alginate-gelatin hybrid gels at 37 °C and 5% CO_2_ for 21 days in presence of chondrogenic media was assessed via a fluorescent based live/dead assay kit (Waltham, MA). Briefly, encapsulated cells were incubated for 30 mins in 200 µl of the solution composed of green-fluorescent dye calcein-AM and red-fluorescent dye ethidium homodimer-1. The solution was then removed, and the gels were washed 3 times with DPBS. The images were captured using Olympus laser scanning confocal microscope (FV1200) and then analyzed to calculate the cell viability. All measurements were conducted in triplicate. Cell viability was measured as a percentage using the following equation:

5 $$\mathrm{Cell Percentage}=100 \times \frac{\mathrm{Live cells }}{\mathrm{Live cells}+\mathrm{Dead cells}}$$

#### Sulfated Glycosaminoglycan Quantification

Sulfated glycosaminoglycan (sGAG) was quantitatively determined using the Glycosaminoglycans Assay kit (Chondrex, Inc., Washington). Using cationic dye 1,9 dimethylmethylene blue (DMB), highly charged sulfated GAGs were bound to DMB and quantitively measured. Per manufacturer’s instructions, 50 µl of 500 µg/ml standard solution was diluted 5 times to get 3.1 µg/ml. 100 µl of standards, DPBS, and samples were added to 96 well plate and then 100 µl of dye solution was added at the top of each well. The plate was then read using a BioTek Eon Microplate reader at 525 nm within 5 mins of adding the dye. sGAG concentration in test samples was calculated using regression analysis.

#### Histological and Immunohistochemical Staining

MSCs encapsulated in spherical alginate and gelatin gels with varying stiffness or cell adhesive properties were stained with 1% (v/v) Hoechst 33342 dye for 5 mins. The gels were then washed with DPBS 3 times and fixed using 1:1 acetone: methanol solution at − 20 °C for 20 mins. After washing with DPBS, the gels were blocked with 5% (w/v) BSA solution for 1 h on a rotator shaker. Gels were then washed 3 times with DPBS and incubated overnight with mouse antibody to human collagen type 2 (1:200) and aggrecan (1:100) at 4 °C. After a triple washing with DPBS, the gels were incubated with Alexa-488 conjugated goat anti-mouse IgG antibody (1:500) for 1 h at 37 °C. The images were captured using Olympus laser scanning confocal microscope (FV1200).

For safranin O staining, the encapsulated gels were washed with DPBS three times before fixing with 1:1 acetone: methanol solution at − 20 °C for 20 mins. Next, the solution was removed and washed again three times with DPBS. Then, the gels were incubated with 0.1% stock solution of safranin O solution for 30 mins at room temperature. The dye solution was removed, and samples washed with distilled water before imaging using a Zeiss Axio ObserverA1 microscope.

### Statistical Analysis

All experiments were conducted at least in triplicates and repeated three times. The data represent the mean ± S.E.M of three independent experiments. Statistical analysis was carried out using one-way ANOVA. The differences between the two sets of data were considered significant at p value < 0.05.

## Results

### Mechanically Tunable Hydrogel Fabrication and Characterization

The hydrogel matrices were fabricated by varying the concentration of alginate from 1.5% to 5.0% (w/v) in the presence or absence of gelatin (concentration was maintained constant at 5.0%). This enabled us to alter the mechanical properties of the gels without affecting the concentration of cell adhesive sites.

**Fig. 2:**
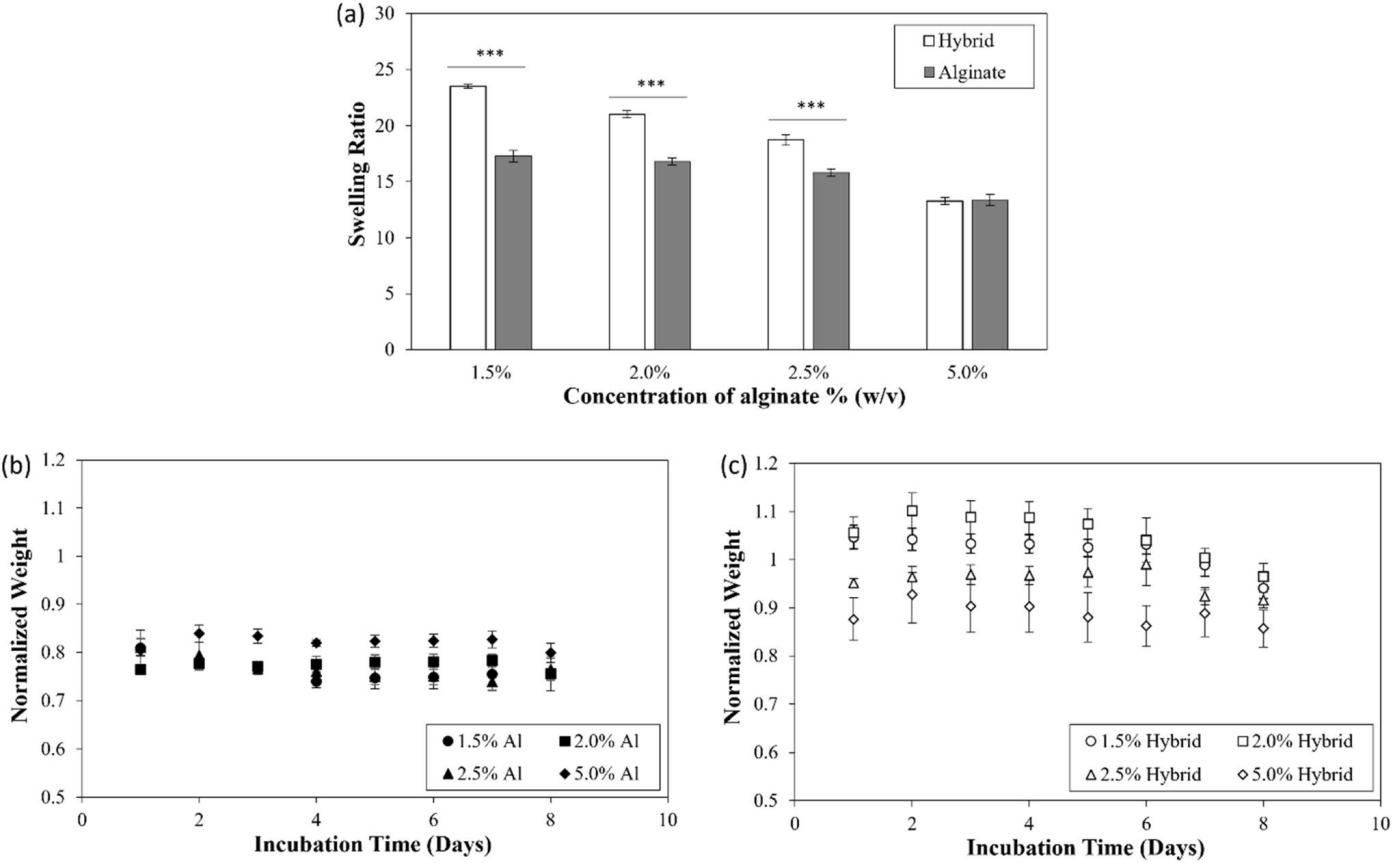
Swelling ratio of alginate hydrogels fabricated with and without gelatin. Concentrations of alginate used were 1.5%, 2.0%, 2.5% and 5.0 % (w/v) with gelatin maintained at 5.0% (w/v) (**a**), degradation of **b** alginate and **c** alginate-gelatin hybrid hydrogels. Data is presented as a ratio of gel weights at different time points to the initial weights. Error bar S.E.M (N = 3), ***p value < 0.001

**Fig. 3:**
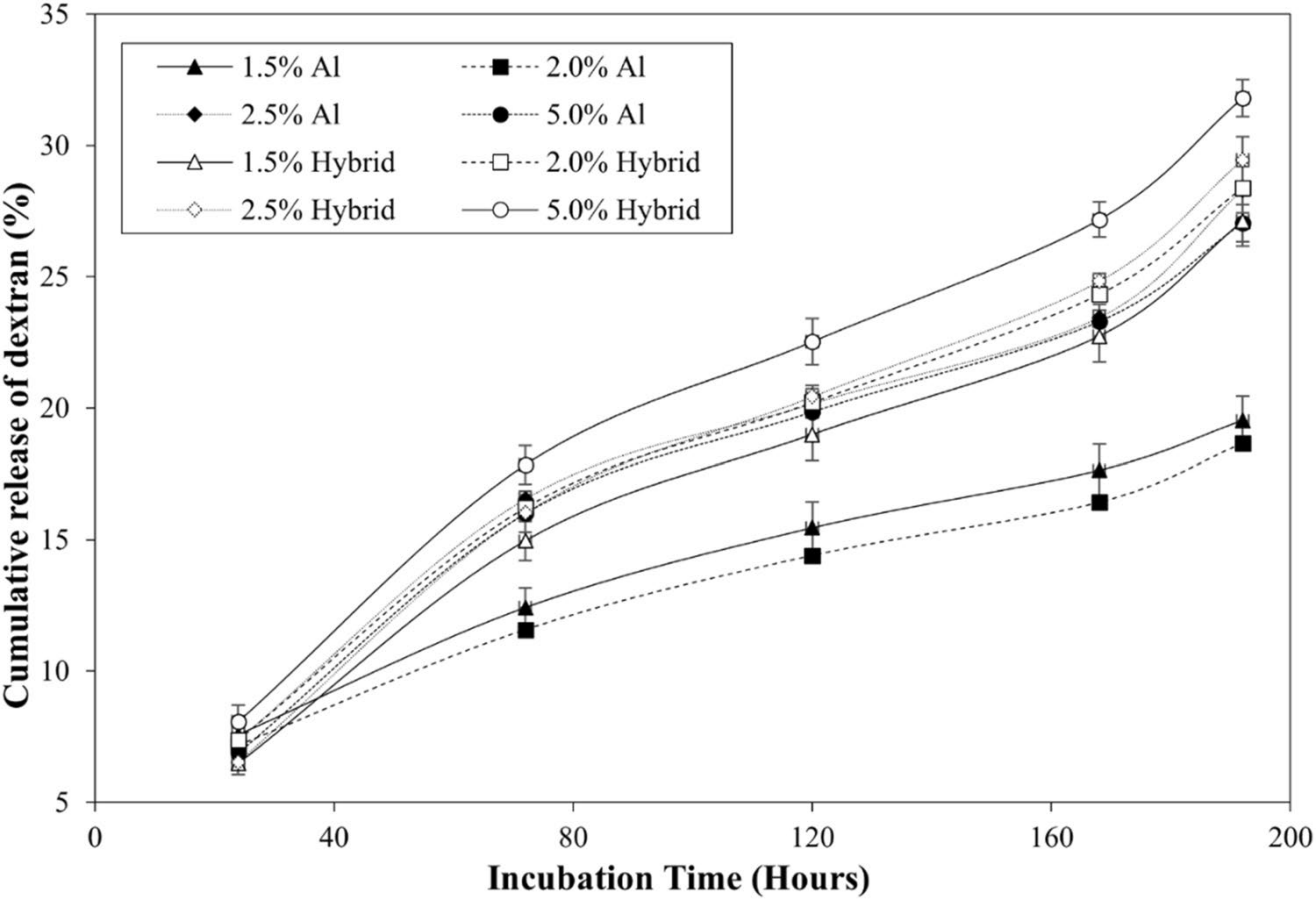
Cumulative release (%) of dextran from alginate and alginate-gelatin (w/v) hybrid hydrogels over an incubation period of 8 days. Error bars S.E.M (N = 3)

The swelling capacity of alginate as well as alginate-gelatin hybrid hydrogels, was analyzed by taking the ratio of the weight of the swollen gels over the dry weight. As observed in Fig. 1a, irrespective of the presence of gelatin, the alginate gels displayed a subtle decrease in swelling ratio (17.3 ± 0.5 for 1.5% to 13.4 ± 0.5 for 5.0% (without gelatin) and 23.5 ± 0.2 for 1.5% to 13.3 ± 0.3 for 5.0% (with gelatin)) with increase in alginate concentrations. Further, it was observed that the incorporation of gelatin increased the swelling ratio of the matrices. However, the increase in swelling ratio in presence of gelatin was more prominent at lower alginate concentrations (p value < 0.001). The integrity of hydrogels was assessed by measuring the rate of degradation of the matrices. For this purpose, the hydrogels were incubated in 2.5 units/ml collagenase type I, and the weights of the gels were measured at different time points. As demonstrated in Figs 2b and c, the degradation ratio (weight of hydrogels at different time points normalized with respect to the initial weight) of alginate hydrogels remained constant for 8 days while the ratio for hybrid gels showed a different trend at the initial time points. The presence of gelatin disrupted the ion crosslinking process between alginate chains, resulting in a more porous network that is both softer and more prone to swelling in DPBS. This effect is more noticeable at lower concentrations of alginate (Fig. 2c). To assess the effect of alginate concentrations on the transport of macromolecules across the gels, the release of FITC-dextran from alginate as well as alginate -gelatin hybrid hydrogels was investigated over a span of 8 days (Fig. 3). For the purpose, the gels were incubated in DPBS free of Ca^2+^ and Mg^2+^ in the absence of collagenase. The concentration of dextran released in the gels at different time points was measured. The observed increased release of macromolecules from hybrid gels compared to the matrices fabricated without gelatin can be correlated to the enhanced swelling of hybrid gels. Further, gelatin is a hydrophilic substance that can dissolve and elute from hydrogel matrices over time. This elution of gelatin can create channels and pathways for the dextran to diffuse more easily, and thus potentially can lead to the increase in the apparent diffusion rate. In addition, we observed a steep increase in release of dextran beyond 160 h, potentially due to the erosion of the gels.

To investigate the mechanism of macromolecule release, the data were fitted to Korsemeyer-Peppas equation and diffusional exponents were calculated (Fig. S1 and Table S1). As observed, the exponent was less than 0.5 for 1.5% and 2.0% alginate gels indicating the transport of macromolecules was primarily by diffusion. The diffusion coefficients were calculated and the diffusivity reduced from (1.6 ± 0.3) × 10^-6^ cm^2^/s to (1.3 ± 0.1) × 10^-6^ cm^2^/s with the increase in alginate concentration from 1.5% to 2.0% (p value < 0.05). However, for 2.5% and 5.0% alginate matrices, the exponent values were greater than 0.5 thereby suggesting that both diffusion and chain relaxation/erosion contributed towards the release of macromolecules. On the contrary, in case of the alginate-gelatin hybrid gels, irrespective of alginate concentration, transport of dextran molecules was via polymer relaxation/erosion.

**Fig. 4:**
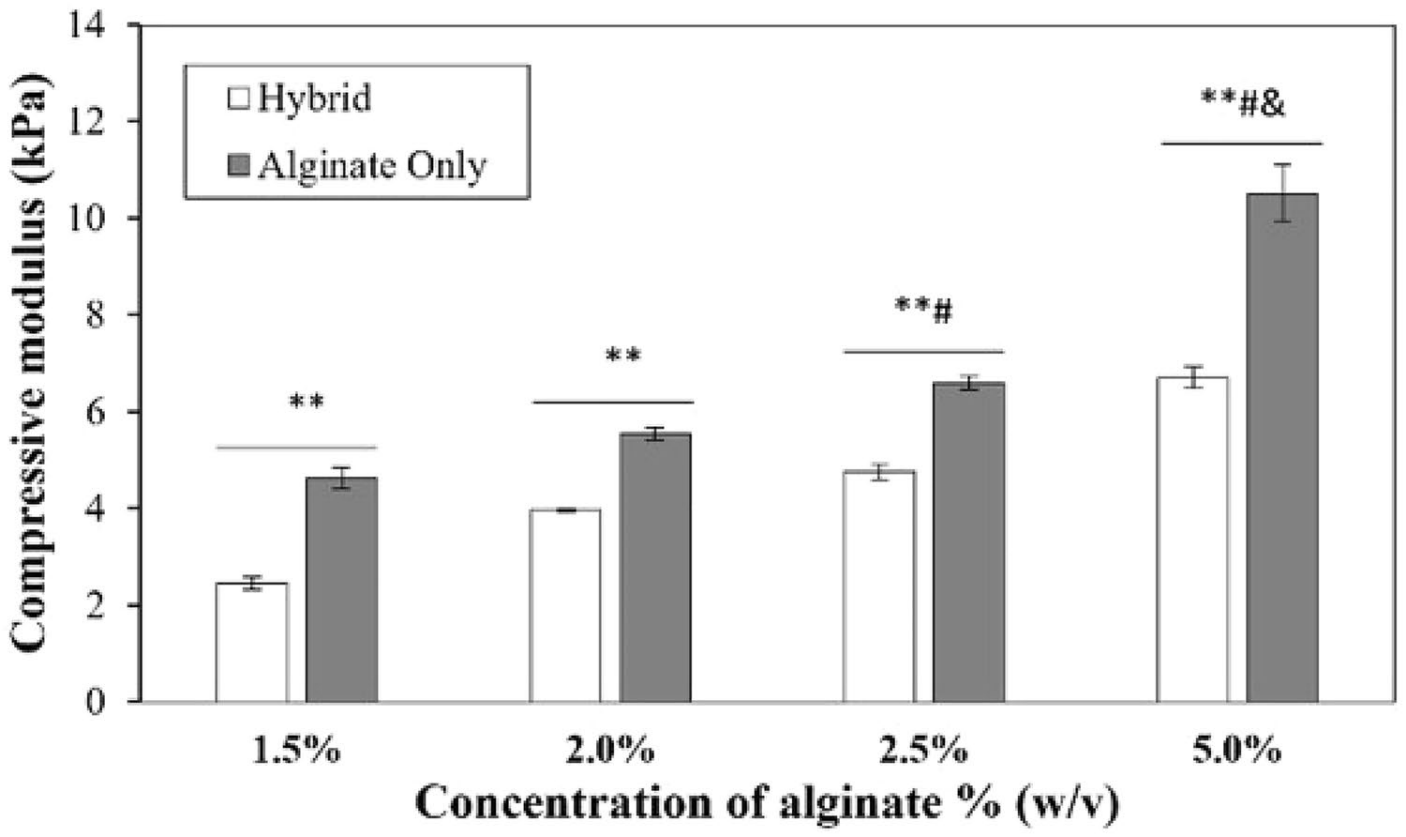
Compressive modulus of alginate and alginate-gelatin hybrid hydrated gels conditions. Error bar S.E.M (N = 3), *p value < 0.05 and **p value < 0.01 with respect to same gel composition (alginate and alginate -gelatin hybrid). ^#^p value < 0.01 with respect to 1.5% and 2.0 % hybrid gels. ^&^p value < 0.01 with respect to 2.5% hybrid hydrogels

The compressive moduli of hydrogels were measured following incubation of the fabricated gels in DPBS for 48 h for hydrated conditions. Irrespective of the presence of gelatin, the compression moduli of the gels increased with an increase in alginate concentrations (p value < 0.05) (Fig 4). However, the moduli of the hydrated hybrid gels were found to be significantly lower than the corresponding alginate gels (p value < 0.05) thereby indicating that the addition of gelatin makes the gels more compliant (Fig 4). The disruption of crosslinking of alginate chains due to the presence of gelatin results in a more porous network in hybrid gels that permit enhanced uptake of solution [28]. This results in hybrid gels being more compliant than alginate gels without gelatin.

### Effect of Alginate Concentrations on Metabolic Activity of Encapsulated MSCs

The influence of alginate concentrations and consequently, the matrix stiffness on the growth of encapsulated MSCs was assessed. The metabolic activity of MSCs encapsulated within alginate hydrogels irrespective of alginate concentration decreased over 21-day culture period (p value < 0.05) (Fig. S2). Further, at any time point, no effect of alginate concentrations on MSC metabolic activity was observed. When MSCs were encapsulated within alginate-gelatin hybrid gels, irrespective of alginate concentration, increase in metabolic activity of MSCs was observed over the span of 15-days. Beyond 15 days, the metabolic activity of MSCs decreased, however, the abatement was not significant for 2.5% and 5.0% alginate-gelatin hybrid gels. Therefore, 2.5% and 5.0% alginate-gelatin hybrid gels were considered for chondrogenic differentiation studies.

### Effect of MSC Passaging History and Gel Stiffness on the Chondrogenic Differentiation of MSCs

**Fig. 5:**
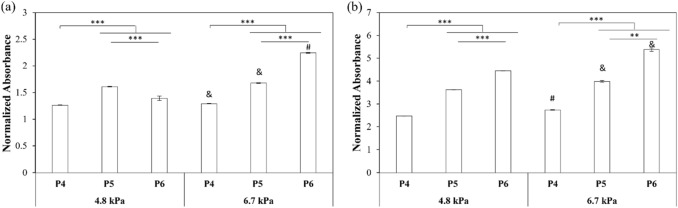
Effect of hybrid gel stiffness and passage number on relative density of MSCs in presence of chondrogenic media for **a** 10 and **b** 21 days. Error bar S.E.M (N = 3). Normalized absorbance refers to ratio of absorbance at any time points to absorbance on day 3. **p value < 0.01 with respect to P5 of same gel composition and ***p value < 0.001 with respect to P4 of same gel composition. ^&^p value < 0.01 and ^#^p value < 0.001 with respect to same passage of different gel composition for the same day

**Fig. 6:**
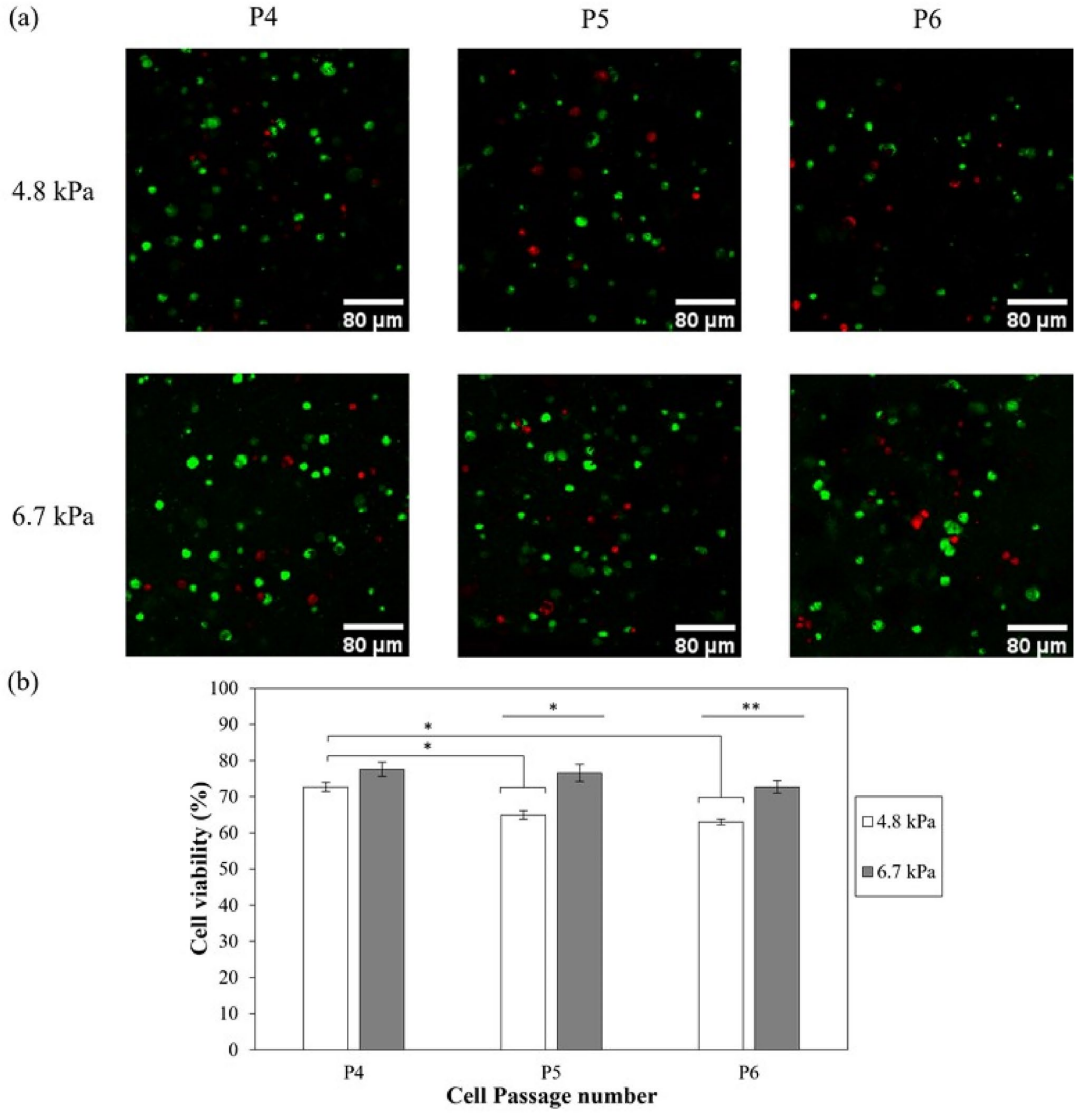
Live/dead staining of differentiated MSCs in 4.8 kPa and 6.7 kPa alginate gelatin gels after 21 days of culture (**a**) and cell viability (%) (**b**) as a function of cell passage number for 4.8 kPa and 6.7 kPa. Error bar S.E.M. (N = 3). *p value < 0.05 and **p value < 0.01. Scale bar = 80 µm

In this study, we explored the interplay between matrix stiffness and passage number of MSCs in promoting chondrogenic phenotype of MSCs. Towards this goal, MSCs (passage number 4-6) were encapsulated within 2.5% and 5.0% alginate-gelatin hybrid gels (4.8 kPa and 6.7 kPa respectively) and incubated in the presence of chondrogenic media. As observed in Figs. 5a and b**,** in case of passage 4 and 5 (P4 and P5), the relative density of MSCs was slightly lower in 4.8 kPa gels than 6.7 kPa gels (p values < 0.01) on days 10 as well as 21 post-encapsulations (p values < 0.01). However, for passage 6 (P6) MSCs, the difference was higher as the relative cell density was much lower in 4.8 kPa gels compared to 6.7 kPa gels **(**Figs 5a and b) (p values < 0.001 for day 10 and p values < 0.01 for day 21). The viability of encapsulated cells was further assessed via fluorescent based live/dead assay kit (Waltham, MA) after 21 days of culture. The confocal images of cells encapsulated within alginate-gelatin hybrid gels confirmed the homogenous distribution of cells throughout the gels (Fig 6a). Quantitative analysis revealed cell passage dependent variation in viability (Fig 6b). The cell viability was found to be decreasing from 72.7 ± 1.3% for P4 to 64.9 ± 1.1% for P5 and 63.0 ± 0.8% for P6 (p value < 0.05) in case of 4.8 kPa hybrid gels. When encapsulated within 6.7 kPa hybrid gels, no statistical difference in viability was observed as a function of passage numbers (p value > 0.05), with the viability varying from 77.6 ± 1.9% for P4 to 76.6 ± 2.4% and 72.7 ± 1.7% for P5 and P6, respectively. Taken together, the results suggest that irrespective of the passaging history, higher metabolic activity was observed with increased matrix mechanics.

**Fig. 7:**
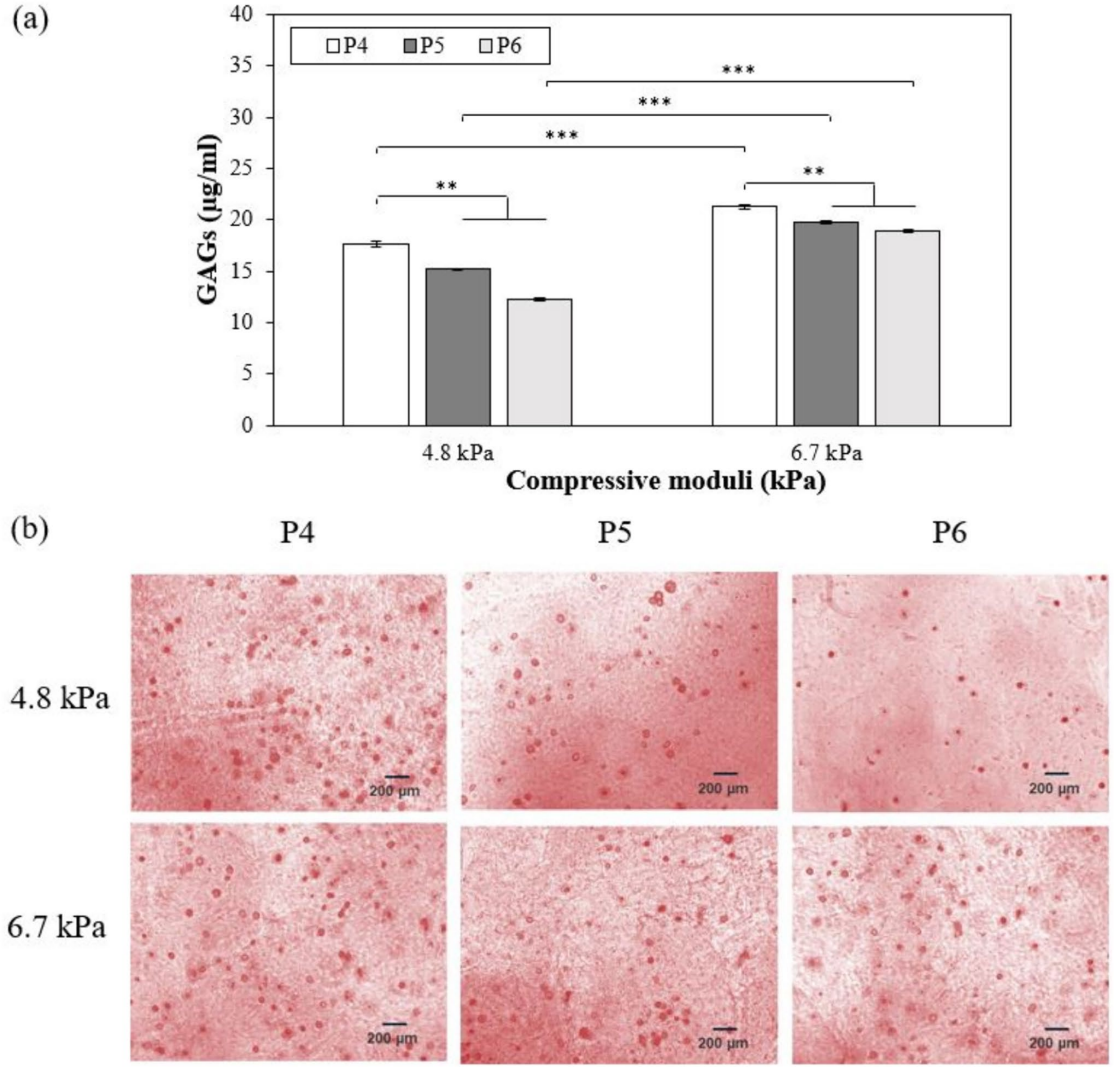
Comparison of the deposition of GAGs as a function of cell passage number for 4.8 kPa and 6.7 kPa (**a**) and safranin-O-staining characterization of the cell-encapsulated hydrogel constructs including 4.8 kPa and 6.7 kPa at day 21 (**b**). Error bar S.E.M. (N = 3). **p value < 0.01 and ***p value < 0.001. Scale bars = 200 µm

**Fig. 8:**
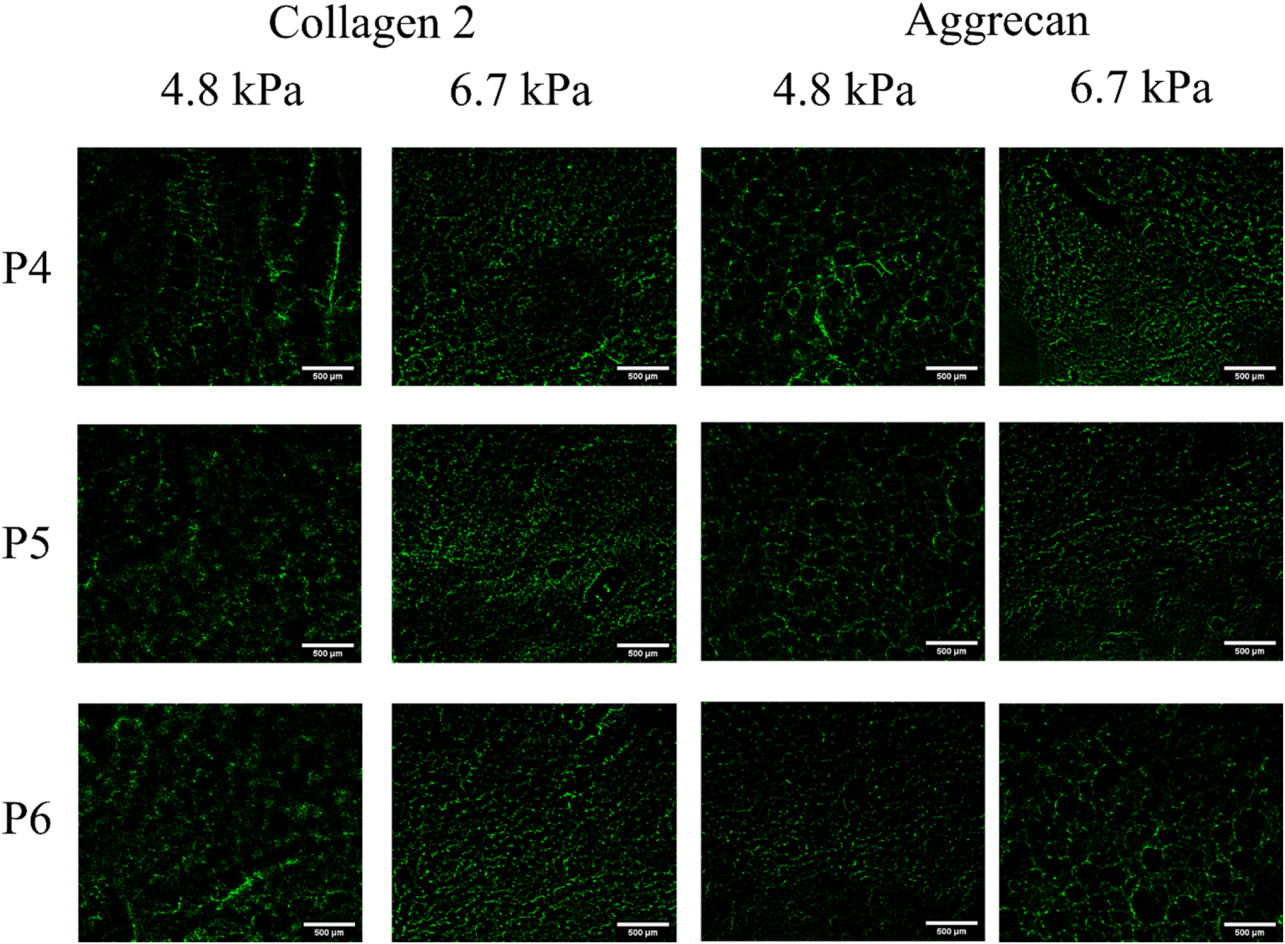
Immunostaining for collagen type 2 and aggrecan at day 21 revealed that both proteins accumulated in all hydrogels. Scale bars = 500 µm

GAGs deposition by MSCs was measured after culture with chondrogenic medium for 21 days while varying stiffness of hybrid gels and passage number of cells. The concentrations of GAGs deposited by MSCs encapsulated within 6.7 kPa alginate-gelatin hybrid hydrogels were higher than those deposited by MSCs within 4.8 kPa gels (p value < 0.001), irrespective of passage numbers (Fig. 7a). It was further observed that P4 MSCs secreted higher concentrations of GAGs in presence of chondrogenic media (17.6 ± 0.3 µg/ml and 21.3 ± 0.2 µg/ml for 4.8 kPa and 6.7 kPa gels, respectively) than P5 MSCs (15.2 ± 0.1 and 19.7 ± 0.2 for 4.8 and 6.7 kPa gels) and P6 MSCs (12.3 ± 0.1 µg/ml and 18.9 ± 0.1 µg/ml for 4.8 kPa and 6.7 kPa, respectively) (p value < 0.001). However, a deeper analysis revealed that matrix mechanics influenced MSC passage-dependent GAG deposition. While 20.7 ± 1.7% increase in GAG deposition was observed upon encapsulation of P4 MSCs in 6.7 kPa hybrid gels vs 4.8 kPa gels, changes were more prominent in P5 and P6 MSCs (30.1 ± 1.2% and 54.4 ± 0.9%, respectively). Further, the analysis also highlighted that GAG deposition by P5 and P6 MSCs was lower than P4 MSCs encapsulated in 4.8 kPa gels by 16.3 ± 1.2% and 43.8 ± 1.9%, respectively compared to 7.8 ± 0.3% and 12.3 ± 1.2%, respectively for 6.7 kPa gels. After 21 days of culture, the constructs were also stained for safranin O to confirm the presence of GAGs (Fig. 7b). As illustrated, increase in passaging history resulted in decrease in GAG deposition. However, increasing the stiffness to 6.7 kPa increased GAG secretion irrespective of MSCs passaging number. Taken together, our observations highlight that altering matrix properties can stimulate MSCs to generate more cartilaginous matrices. The interplay between matrix mechanics and MSC passaging history on chondrogenic commitment was further evaluated by assessing the expression of chondrogenic markers including collagen type 2 and aggrecan. Immunostaining for collagen type 2 and aggrecan at day 21 showed that these specific cartilaginous ECM proteins were expressed in both hydrogels irrespective of MSC passages (see Fig. 8). Moreover, as shown in Fig. 8, a more uniform spatial distribution of deposited collagen type 2 was observed in P6 MSCs than P4 MSCs in 4.8 kPa gels. On the other hand, no such difference in collagen 2 deposition was noticed between the 3 passages in the stiffer gels. In case of aggrecan, P4 deposited more than P6 in 4.8 kPa gels. When the cells were encapsulated within 6.7 kPa gels, similar trend was observed for aggrecan. Therefore, the increase in matrix stiffness stimulated MSCs to deposit cartilaginous matrix independent of MSC passages.

## Discussion

Improved understanding of putative contributions of mesenchymal stem cells (MSCs) to cell therapy and regenerative medicine have resulted in widespread investigation for autologous as well as allogenic applications for various diseases. But this requires large number of cells [29]. However, studies have suggested decrease in robustness of MSCs manifested by reduced replication and multi-lineage differentiation capability with increasing passage number [30]. As a result, most of the clinical applications utilize MSCs with a low *in vitro* passage number of less than 5 [31]. However, such low passage might not yield enough cells for MSC-based therapy. Thus, the goal of this study was to explore the effect passaging history on chondrogenic differentiation potential of MSCs. A recent study explored the effect of matrix stiffness and culture dimensionality (2D vs 3D culture) on chondrogenesis of MSCs [32]. It reported that irrespective of dimensionality soft/compliant gels (~ 1 kPa) have been shown to promote chondrogenesis of MSCs [32] as manifested from increased expression of chondrogenic markers SOX9, ACAN, and COL2. This further correlated with increased SMAD2/3 nuclear localization and MSCs condensation. To understand the interplay between matrix stiffness and composition in inducing MSC chondrogenesis, hydrogels with two stiffnesses (7.5 kPa and 36 kPa) were fabricated with physiologically relevant concentrations (0-10%) of chondroitin sulfate or heparan sulfate [33]. The study reported that chondroitin sulfate at lower mechanical stiffness enhanced chondrogenic differentiation of MSCs. Stiffer hydrogels (36 kPa), irrespective of chemical composition, inhibited neocartilage formation [33]. On the other hand, other studies using murine chondrocytes and ATDC5 reported that stiffer matrix environment (~ 500 kPa) promote chondrogenic phenotype [34]. Similarly, while exploring the combinatorial effect of matrix stiffness and adhesion ligand density on chondrogenic differentiation of MSCs, it was reported that higher ECM adhesivity and matrix stiffness (25 kPa) promoted chondrogenic differentiation of MSCs [35]. Thus, these studies highlight that while matrix stiffness is a regulator of chondrogenesis of MSCs, its exact role is still not completely understood. Hence, the interplay between matrix compliance and passaging history on chondrogenic differentiation of mesenchymal stem cells encapsulated within alginate-gelatin hybrid hydrogels was extensively investigated in this paper.

The effect of alginate concentrations on swelling ratio, degradation ratio, and compressive modulus of alginate as well as alginate-gelatin hybrid gels was tested. The swelling ratio was found to decrease for both alginate only and hybrid gels which attributed to the reduced water uptake with the increase in polymer concentration. Further, it was observed that the incorporation of gelatin improved the swelling ratio of the matrices, possibly due to the hydrophilicity of gelatin. 1.5% and 2.0% alginate hydrogels had the lowest diffusivity, and this is related directly to the highest water uptake of gels. However, the trend for the 5.0% hybrid gels was different as despite of the lowest swelling ratio, the diffusivity was the highest due to the relaxation and erosion of gels which increased the release of macromolecules. The compressive moduli of alginate gelatin hybrid gels were significantly higher than the matrices fabricated with alginate only due to the high polymer concentration as well as densification of alginate/gelatin strands upon drying.

Our study revealed that alginate-gelatin matrices promoted the deposition of cartilaginous matrices irrespective of passaging history of MSCs. The relative deposition of GAGs, as reflected from Safranin-O-staining and DMB quantification, decreased with increase in passage number from 4 to 6, irrespective of matrix mechanics. These observations are in agreement with other studies that reported that the chondrogenic commitment reduced at higher passages [36]. However, in this study we observed that increasing matrix stiffness facilitated improved GAG deposition by P6 MSCs, thereby suggesting the potential role of matrix mechanics in rescuing the reduced differentiation capability of these cells. In our studies, we did not observe significant difference in collagen type II, the fibrillar collagen found in cartilage, deposition as a function of MSC passaging. As a matter of fact, we observed a subtle increase in collagen deposition in P6 MSCs in the compliant gels compared to P4 MSCs. This is inconsistent with previous study that established reduced Col2A gene expression by MSCs with higher passage number [37]. The observed difference can potentially be attributed to the difference in the source of MSCs; in this study bone marrow derived MSCs as opposed to adipose MSCs used in the previous study.

In summary, we compared the effects of encapsulating human bone marrow derived-MSCs within alginate gelatin hybrid gels of different compliances. While MSCs with lower passaging history display increased chondrogenic commitment, our proof-of-concept study demonstrated that altering matrix properties can stimulate higher passaged MSCs to generate more cartilaginous matrices.

### Supplementary Information

Below is the link to the electronic supplementary material.

Supplementary file1 (PDF 329 KB)
