# Supplementary material for: Investigating the Interplay Between Matrix Compliance and Passaging History on Chondrogenic Differentiation of Mesenchymal Stem Cells Encapsulated Within Alginate-Gelatin Hybrid Hydrogels
Source: Ann Biomed Eng. 2023 Jul 15;51(12):2722–34. doi: 10.1007/s10439-023-03313-y (PMC10632279; doi:10.1007/s10439-023-03313-y)
Supplement: Supplementary file 1 — Supplementary file1 (PDF 329 KB) [file 10439_2023_3313_MOESM1_ESM.pdf]

**Table S1.** Calculated values of diffusional exponent (n) of hydrogels. Exponent is presented as average of three hydrogel results ± error.

| Alginate concentration % (w/v) | Alginate gels | Alginate-gelatin hybrid gels |
|---|---|---|
| 1.5% | 0.449 ± 0.011 | 0.949 ± 0.012 |
| 2.0% | 0.454 ± 0.004 | 0.930 ± 0.018 |
| 2.5% | 0.529 ± 0.024 | 0.967 ± 0.011 |
| 5.0% | 0.596 ± 0.029 | 0.969 ± 0.014 |

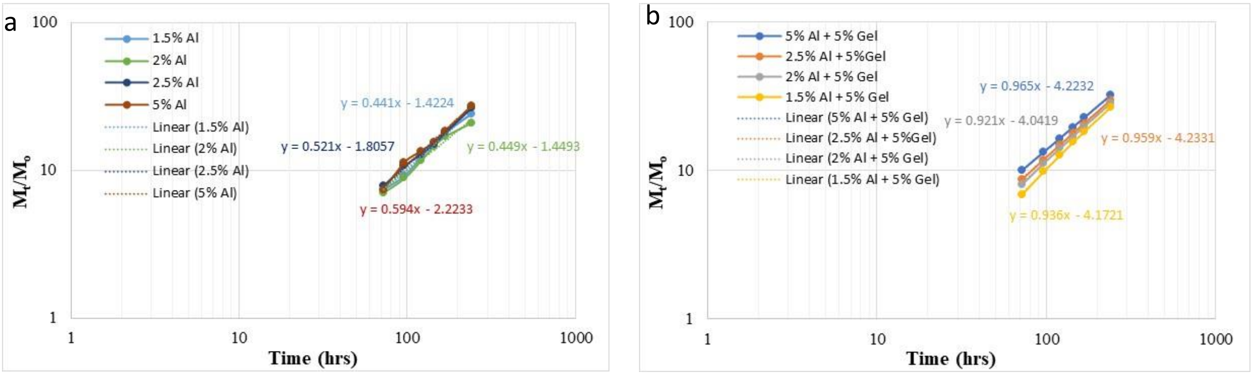


**Figure S1.** Log-log plot for Mt/Mo for (a) alginate only and (b) alginate with 5.0% Gelatin

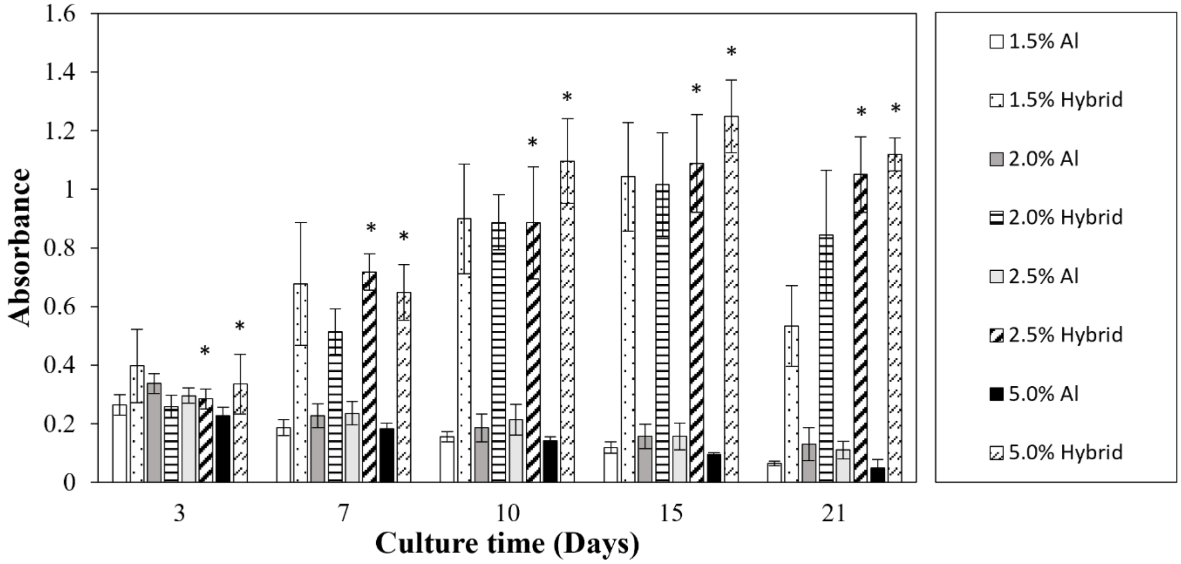


**Figure S2.** Effect of alginate and alginate-gelatin hybrid gels compositions on metabolic activity of MSCs over a span of 21 days. Error bar S.E.M (N=3). *p-value <0.05 with respect to 2.0% alginate only.
